# Functional and Genomic Evidence of L-Arginine-Dependent Bacterial Nitric Oxide Synthase Activity in *Paenibacillus nitricinens* sp. nov.

**DOI:** 10.3390/biology14060733

**Published:** 2025-06-19

**Authors:** Diego Saavedra-Tralma, Alexis Gaete, Carolina Merino-Guzmán, Maribel Parada-Ibáñez, Francisco Nájera-de Ferrari, Ignacio Jofré-Fernández

**Affiliations:** 1 Scientific and Technological Bioresource Nucleus (BIOREN), Universidad de La Frontera, Avenida Francisco Salazar 01145, P.O. Box 54-D, Temuco 4811230, Chile; d.saavedra05@ufromail.cl (D.S.-T.); francisco.najera@ufrontera.cl (F.N.-d.F.); 2 Laboratory of Geomicrobiology, Department of Chemical Sciences and Natural Resources, Universidad de La Frontera, Avenida Francisco Salazar 01145, P.O. Box 54-D, Temuco 4811230, Chile; carolina.merino@ufrontera.cl; 3 Millennium Institute Center for Genome Regulation (CGR), Valenzuela Puelma 10207, La Reina, Santiago 7860006, Chile; alexis.gaete@inta.uchile.cl; 4 Laboratory of Rhizobiology and Microbiology, Facultad de Ciencias Agropecuarias y Medioambiente, Universidad de La Frontera, Avenida Francisco Salazar 01145, P.O. Box 54-D, Temuco 4811230, Chile; maribel.parada@ufrontera.cl

**Keywords:** aerobic NO production, bacterial nitric oxide synthase, L-arginine metabolism, nitric oxide

## Abstract

Although nitric oxide (NO) production in bacteria has traditionally been associated with denitrification or stress responses in model or symbiotic organisms, functionally validated L-arginine-dependent nitric oxide synthase (bNOS) activity has not been documented in free-living, non-denitrifying soil bacteria. This paper reports *Paenibacillus nitricinens* sp. nov., a bacterium isolated from rainforest soil capable of synthesizing NO via a bNOS under aerobic conditions. A *bnos*-specific PCR confirmed gene presence, while whole-genome sequencing (6.7 Mb, 43.79% GC) revealed two nitrogen metabolism pathways, including a *bnos*-like gene. dDDH (<70%) and ANI (<95%) values with related *Paenibacillus* strains support the delineation of this isolate as a distinct species. Extracellular and intracellular NO measurements under aerobic conditions showed a dose-dependent response, with detectable production at 0.1 µM L-arginine and saturation at 100 µM. The addition of L-NAME reduced NO formation, confirming enzymatic mediation. The genomic identification of a *bnos*-like gene strongly supports the presence of a functional pathway. The absence of canonical nitric oxide reductase (*Nor*) genes or other typical denitrification-related enzymes reinforces that NO production arises from an alternative, intracellular enzymatic mechanism rather than classical denitrification. Consequently, *P. nitricinens* expands the known repertoire of microbial NO synthesis and suggests a previously overlooked source of NO flux in well-aerated soils.

## Simple Summary

Bacteria are known to influence the nitrogen cycle in soils, often through processes that require low oxygen, such as denitrification. However, this paper describes a newly discovered bacterium, *Paenibacillus nitricinens*, which produces nitric oxide (NO), a reactive nitrogen compound, even in the presence of oxygen. This NO production is driven by an enzyme that uses the amino acid L-arginine, resembling a mechanism found in animals but rarely confirmed in soil bacteria. The bacterium was isolated from Chilean rainforest soil and showed a strong ability to grow using different carbon and nitrogen sources. Genetic analyses confirmed that it is a distinct species and revealed a gene responsible for NO production. Laboratory experiments demonstrated that this bacterium emits NO in a dose-dependent manner when given L-arginine, and this effect is blocked by a specific enzyme inhibitor, proving the process is enzyme-driven. These findings suggest that NO could be produced in well-aerated soils through non-canonical mechanisms, highlighting a previously overlooked microbial contribution to the nitrogen cycle. This discovery expands our understanding of how microbes regulate nutrients in natural ecosystems and may open new possibilities for uncovering mechanisms that control nutrient flow in temperate forest soils.

## 1. Introduction

Nitric oxide (NO) is a reactive nitrogen compound that mediates oxidation–reduction processes and antimicrobial defense in many soils [1]. It also acts as a signaling molecule, regulating microbial interactions, microbial networks, plant–microbe relationships, and biofilm formation, facilitating bacterial communication [2,3]. Denitrification and nitrification are well-documented pathways for NO synthesis in soils, primarily linked to the nitrogen cycle and regulated by oxygen availability [4,5]. However, an alternative NO-producing mechanism has been proposed, involving L-arginine-dependent nitric oxide synthase (bNOS), which functions independently of nitrate, nitrite, or ammonium limitations [6,7]. Despite this pathway being of potential ecological importance, especially under aerobic conditions, its presence and functional validation in native, non-symbiotic, free-living bacteria from complex natural environments are largely lacking. Most of the available evidence arises from genetic or in vitro studies using heterologous systems rather than direct biochemical characterization in wild isolates [8,9].

Several bacteria, such as *Bacillus subtilis* and *Pseudomonas aeruginosa*, have demonstrated NO production under aerobic conditions through bNOS or partial denitrification enzymes [10,11]. However, these cases are mostly limited to stress-related physiology or pathogenicity. In contrast, environmental isolates, particularly those from complex soil matrices, remain largely unexplored for aerobic, L-arginine-dependent NO biosynthesis.

The genus *Paenibacillus* displays broad ecological distribution and metabolic versatility, including nitrogen fixation, nitrate reduction, and various enzymatic pathway applications [12,13,14]. Nonetheless, NO production via a bNOS pathway in this group has not been functionally demonstrated, despite some taxa using NO as an intermediate during denitrification [15]. While genomic predictions have hinted at the existence of *bnos*-like genes beyond the *Bacillus* lineage, their transcriptional or enzymatic activity remains unproven in environmental *Paenibacillus* isolates [16]. Also, environmental parameters such as oxygen availability, moisture, metal cofactors, and redox fluctuations may influence bNOS activity in soils globally [4,5]. This represents a major knowledge gap in understanding the nitrogen-processing capabilities of this ecologically significant genus.

Temperate rainforest soils, such as those in southern Chile, offer a unique ecological backdrop: high organic nitrogen content, minimal inorganic N inputs, and seasonal oxic–anoxic transitions [17,18,19]. These conditions support facultative, oxygen-tolerant bacteria like *Paenibacillus* spp. [13], which could exploit amino-acid-derived nitrogen pathways for NO synthesis. Such possibilities align with recent views proposing NO as a cryptically cycled intermediate beyond canonical denitrification routes [20].

To address these gaps, this paper reports the isolation and characterization of a novel *Paenibacillus* strain from Chilean rainforest soil, capable of aerobic, L-arginine-dependent NO production. This work provides the first functional evidence of bNOS activity in a free-living, wild-type *Paenibacillus* strain. Genomic, phylogenetic, and functional analyses support the presence of a bNOS-mediated NO synthesis pathway. These findings contribute to our understanding of non-canonical nitrogen cycling in aerobic soil environments.

## 2. Materials and Methods

### 2.1. Sampling Site, Soil Chemical Characterization, and Bacterial Isolation

Four composite soil samples were collected from the upper 15 cm (Ah horizon) in Alerce Costero National Park, Chile (40°12′ S, 73°26′ W). These soils were derived from metamorphic mica schists with illite–kaolinite clays [21] and showed minimal anthropogenic disturbance. One portion of each sample was sieved (<2 mm) and stored at 4 °C for isolation. The other was air-dried for chemical analysis (Table 1). Soil pH was measured in 1:2.5 (*w*/*v*) soil-to-deionized water suspensions using a calibrated pH meter (PHD 1, PCE instruments, Spain). Total carbon (C) and total nitrogen (N) contents were determined using a TOC analyzer (TOC-VCSH, Shimadzu, Japan) and the Kjeldahl digestion method (UDK149, Velp, Italy), respectively. Inorganic nitrogen species, including nitrate (NO_3_^−^) and ammonium (NH_4_^+^), were quantified via vanadium reduction and salicylate-based colorimetric assays [22,23]. Soluble iron (Fe^2+^) was extracted in 0.5 M HCl and quantified using the ferrozine assay (λ = 562 nm), while total Fe was measured after hydroxylamine reduction, and Fe^3+^ was calculated as the difference [24]. A 10 g soil subsample was homogenized in 0.85% NaCl solution, serially diluted (10^−1^ to 10^−9^), and plated (200 μL) on Plate Count Agar (BD, Heidelberg, Germany) at pH 7.0. The plates were incubated aerobically at 30 °C for 48 h. Individual colonies were isolated and purified by repeated streaking. All subsequent experiments were performed with five independent biological replicates, and each was measured in triplicates. 

### 2.2. 16S rRNA Sequencing of Isolates

Genomic DNA was extracted from the isolates using the PowerSoil DNA isolation kit (Qiagen, Hilden, Germany). The V1–V9 region of the 16S rRNA gene (~1500 bp) was amplified (SapphireAmp Fast PCR Master Mix, Takara Bio, San Jose, CA, USA) using primers 27F (5′-AGA GTT TGA TCM TGG CTC AG-3′) and 1492R (5′-CGG TTA CCT TGT TAC GAC TT-3′) and the following thermal profile: 94 °C for 10 s, 30 cycles of 94 °C for 1 min, 55 °C for 5 s, and 72 °C for 10 s, followed by 72 °C for 5 min. The amplicons were purified (EXOSap-IT Express, Applied Biosystems, Foster City, CA, USA) and sequenced (ABI 3500, Applied Biosystems, CA, USA). The nucleotide sequences were trimmed, compared to GenBank, and aligned using the MEGA software (v12). Species with <98% identity were retained for phylogenetic analysis (LPSN). Neighbor-joining and maximum likelihood trees were applied to the Kimura two-parameter model. Bootstrap (1000 replicates) was used to test the robustness of the tree.

### 2.3. Design and Amplification of bnos-Targeting Primers

Primers targeting the *bnos* gene were designed in silico and experimentally validated to guide sample selection for *in vitro* studies. A GenBank search for “nitric oxide synthase” in bacteria yielded sequences (Supplementary Methodology S1 and Table S1), which were analyzed using the NGphylogeny.fr web service [27] in advanced mode (https://github.com/C3BI-pasteur-fr/ngphylogeny-galaxy.git, accessed on 12 January 2025). The selected workflow included multiple alignment with MAFFT (v7.047_1), alignment curation with BMGE (1.12_1), tree inference with PhyML (v3.3_1), and tree rendering with JalView (v2.11.4.1). Thirteen *Bacillus* and eleven *Paenibacillus* species with high sequence homology were selected (Figures S1 and S2, Table S2). Strains with no *bnos* homologs (e.g., AC2 and AC5) were excluded. A consensus region was used to design the primers following strict criteria. The full details of the design and validation processes are available in Supplementary Methodology S1. The selected primer pair bNOSPF1 (5′-CCCTTTAACGGCTGGTATATGG-3′) and bNOSPR1 (5′-TATGATGGTCAACGATGCTGAC-3′) amplified a 211 bp fragment. PCR was performed using the SapphireAmp^®^ Master Mix (Takara Bio, CA, USA), under optimized conditions (annealing at 57 °C). Only strain AC7 yielded a product of the expected size, which was purified, Sanger-sequenced, and confirmed by blastn/blastx searches, with and without genus restriction.

### 2.4. Hybrid Whole-Genome Sequencing and Assembly

Whole-genome sequencing of *Paenibacillus* sp. strain AC7 was carried out using a hybrid strategy combining Oxford Nanopore long-read sequencing (SQK-RBK114.96 kit; R10.4.1 FLO-MIN114 flow cell, Oxford, UK) and Illumina short-read sequencing (Nextera XT Library Prep Kit; NovaSeq 6000 platform, Oakland, CA, USA), performed by MicrobesNG (Birmingham, UK). Basecalling and demultiplexing were performed according to the facility’s pipeline. Quality control of long reads was assessed using NanoPlot v1.41.0 (https://github.com/wdecoster/NanoPlot, accessed on 12 January 2025) while Illumina reads were filtered and trimmed using default parameters in QIIME 2 v2024.10 (https://qiime2.org/, accessed on 12 January 2025) [28]. Error correction and chimera removal were performed using the DADA2 pipeline v1.26 (https://benjjneb.github.io/dada2/, accessed on 12 January 2025) [29]. Hybrid de novo genome assembly was conducted using Flye v2.9.6 (https://github.com/fenderglass/Flye, accessed on 12 January 2025), followed by polishing with Pilon v.1.14 (https://github.com/broadinstitute/pilon, accessed on 12 January 2025). Assembly quality was assessed with BUSCO v5.5.0 (https://busco.ezlab.org/, accessed on 12 January 2025) [30] using the bacillales_odb10 lineage dataset. Genome annotation was performed using Prokka v1.14.6 (https://github.com/tseemann/prokka, accessed on 12 January 2025), and protein functions were further refined using eggNOG-mapper v5.0.2 (http://eggnog-mapper.embl.de/, accessed on 12 January 2025), based on orthology assignment from eggNOG KEGG orthology assignments were performed using KofamKOALA v2025-05-01 (https://www.genome.jp/tools/kofamkoala/, accessed on 12 January 2025), allowing identification of gene pathways associated with nitrogen metabolism. Additional pathway inference was supported by Flux Balance Analysis (FBA) using the ModelSEED database v2.2.1 (https://github.com/ModelSEED/ModelSEEDDatabase, accessed on 12 January 2025). Antimicrobial resistance genes were screened using StarAmr v0.11.0 (https://github.com/phac-nml/staramr, accessed on 12 January 2025) and secondary metabolite biosynthetic gene clusters were identified via antiSMASH v6.1.1 (https://antismash.secondarymetabolites.org/, accessed on 12 January 2025).

Taxonomic assignment was confirmed using digital DNA–DNA hybridization (dDDH) through the Type Strain Genome Server (TYGS) [31] v400 (https://tygs.dsmz.de, accessed on 12 January 2025). Phylogenomic relationships were reconstructed using Genome Blast Distance Phylogeny (GBDP) v3.0, and trees were assembled using FASTME v2.1.6.1 (http://www.atgc-montpellier.fr/fastme/, accessed on 12 January 2025), with 1000 bootstrap replicates. Comparative analyses with *Paenibacillus* reference genomes were performed using OrthoANI (OAT) v0.93.1 and GGDC v3.0 for dDDH estimation [32] (https://ggdc.dsmz.de/distcalc2.php, accessed on 12 January 2025). The complete genome of strain AC7 was deposited in GenBank under BioProject PRJNA1233252, BioSample SAMN47264457, and TaxID 3367691.

### 2.5. Phenotypic Characterization

Bacterial samples were prepared for observation under high-vacuum scanning transmission electron microscopy (HV-STEM, SU3500 HITACHI, Tokyo, Japan). Cultures were centrifuged at 8000× *g* for 5 min at room temperature, washed twice with sterile water, and resuspended in water. A 50 µL drop of the suspension was placed onto a carbon-coated grid (200 mesh) and incubated for 5 min. The grid was then stained with 2% aqueous uranyl acetate for 2 min to enhance contrast, followed by a brief rinse in sterile water to remove excess stain. Residual liquid was carefully blotted with filter paper. Samples were imaged under high-vacuum conditions using an accelerating voltage of 30 kV, a working distance of 5 mm, and a spot size of 50–60. Imaging was performed using a standard secondary electron (SE) detector. Cell motility was determined using semi-solid TTC motility agar (stab method, 35 °C, 18 h); Gram staining followed the standard crystal violet–iodine–ethanol–safranin protocol and endospore formation was assessed using the Schaeffer–Fulton method with malachite green and safranin counterstain. Also, biochemical assays included oxidase, catalase, citrate utilization, urease, indole production, Voges–Proskauer, methyl red, and the fermentation of glucose, lactose, and mannitol test were included according with Chauhan and Jindal [33].

MALDI-TOF MS identification was performed using a modified protein extraction protocol [34]. Fresh bacterial culture was washed with sterile water, resuspended in 70% ethanol, and centrifuged (10,000× *g*, 3 min). The pellet was then treated with 20 μL of formic acid, followed by 20 μL of acetonitrile. After vortexing and a second centrifugation (10,000× *g*, 3 min), 1 μL of the supernatant was spotted onto a steel target plate, air-dried, and overlaid with 1 μL of a saturated solution of α-cyano-4-hydroxycinnamic acid (HCCA) prepared in 50% acetonitrile with 2.5% trifluoroacetic acid. Mass spectra were acquired using a MALDI-TOF MS Autoflex Speed instrument (Bruker Daltonics, Bremen, Germany) in linear positive mode (20 kV acceleration voltage, 220 ns extraction delay), averaging 1200 laser shots across the 2000–20,000 *m*/*z* range. Spectra were processed with MALDI Biotyper Compass 4.1 and compared to the Bruker bacterial reference library within the 2000–12,000 m/z range. Identification scores > 1.7 were accepted for genus-level assignment, and >2.0 for species-level identification, in accordance with the manufacturer’s recommendations.

Nitrogen fixation and phosphorus solubilization were qualitatively assessed. For nitrogen fixation, cultures were grown on Burk’s nitrogen-free agar [35], and colony development indicated a positive result. Phosphorus solubilization was tested using SMRS1 agar, with a clear halo around colonies confirming its activity [36].

### 2.6. Growth and Nutrient Preference

Temperature and pH tolerance were evaluated in Zobell Marine Broth (2216, HiMedia, Kennett, PA, USA). For temperature tolerance, 9 mL of culture was incubated at 4 °C, 12 °C, 20 °C, 30 °C, and 40 °C with constant shaking, and OD_600_ was measured after 48 h. For pH tolerance, the medium was adjusted to pH 4.5–8.5 (0.5-unit increments) using HCl/NaOH, incubated at 30 °C (170 rpm), and OD_600_ was measured after 48 h using a Feyond A300 microplate reader (Hangzhou, China). Carbon and nitrogen utilization were assessed using a microplate-based assay.

For carbon utilization, 220 µL reactions contained 150 µL basal medium (2 g/L NH_4_Cl, 0.01 M potassium–phosphate buffer pH 7.0, 10 mL/L modified Wolin’s mineral solution), 20 µL AC7 inoculum (OD_600_ = 0.1), and 50 µL of the carbon source (20 mM). The carbon sources tested included fumarate, pyruvate, dextrose, glycerol, L-arginine, citric acid, L-alanine, D(−)-ribose, D(+)-galactose, L-glutamine, α-ketoglutaric acid, γ-aminobutyric acid, L-lysine, oxalic acid, D-mannitol, L-glutamic acid, D(−)-fructose, D(+)-trehalose, D(+)-glucose, succinic acid, acetate, malic acid, L(+)-arabinose, L-cysteine, sucrose, formate, L-ascorbic acid, thiamine, folic acid, pyridoxine, and 4-aminobenzoic acid. A control group without any treatment was included in the study. For nitrogen utilization, 220 µL reactions contained 100 µL nitrogen-free basal medium, 20 µL AC7 inoculum, 50 µL nitrogen source (2 g/L, e.g., protease peptone, yeast extract, urea, NH_4_Cl, and NaNO_3_), and 50 µL (20 mM) of glucose, sucrose, or fructose to assess carbon dependence. Controls without carbon or nitrogen were also included. OD_600_ was measured after 48 h and converted to CFU/mL using a standard curve generated from exponential growth data (OD_600_ recorded every 10 min; CFU determined by plating every 30 min). All experiments were assessed in quintuplicate.

### 2.7. Extracellular NO Measurement

NO production from L-arginine catalysis was measured under aerobic conditions using an amperometric NO probe (ISO-NOP) connected to a radical analyzer (TBR1025 and Apollo 1000; World Precision Instruments, Sarasota, FL, USA) [37] with some modifications to the bacterial solutions. Experiments were performed in triplicate in a four-port closed chamber (NOCHM-4, World Precision Instruments, FL, USA) at 20 °C with constant low-speed stirring. To minimize NO diffusion while maintaining aerobic conditions, the chamber was partially sealed with a cap containing a 0.5 mm hole. Oxygen availability was monitored using an oxygen probe (ISO-OXY-2), which showed no significant variation during incubation. Each experiment used 3 mL of the bacterial culture (nutrient broth, OD_600_ = 0.1). The baseline stabilization lasted 20 min. L-arginine was added stepwise (0.1, 1, 5, 20, 100, and 1000 µM) [8] every 10 min or until the signal plateaued. The inhibitor NG-nitro-L-arginine methyl ester (L-NAME, 200 µM), a widely used NOS-specific competitive inhibitor [38], was added at the end to confirm the enzymatic nature of NO production. A standard curve with S-Nitroso-N-Acetylpenicillamine (SNAP) and CuCl_2_ was used to convert the voltage signal to µM NO. The recorded voltage (mV) was converted to NO concentration (µM) using a standard calibration curve based on SNAP dissociation in CuCl_2_. Data acquisition was performed using LabTrax v24 (iWorks, NH, USA).

### 2.8. Intracellular NO Detection

Intracellular NO production was assessed using the fluorescent probe DAF-FM (D23844; Invitrogen, OR, USA) following bacterial adaptation [39,40]. The AC7 cultures (OD_600_ = 0.1) were incubated with 10 µM DAF-FM in PBS (pH 7.2) for 30 min, resuspended in nutrient broth, and aliquoted (200 µL) into microplate. L-arginine was added at final concentrations of 1, 5, 20, 100, and 1000 µM. Fluorescence emission was recorded every 5 min at 20 °C under constant agitation using a Feyond A300 microplate reader (Hangzhou, China). Controls without L-arginine were included to subtract baseline fluorescence and normalized to baseline (F/F_0_ ratio).

### 2.9. Statistical Analysis

All quantitative data were analyzed using GraphPad Prism v10.0. One-way analysis of variance (ANOVA) was used to evaluate the differences between treatments (e.g., carbon and nitrogen utilization, and NO production). Prior to ANOVA, data normality was verified using the Shapiro–Wilk test, and homogeneity of variances was assessed using Levene’s test. When significant differences were found (*p* < 0.05), Tukey’s honest significant difference (HSD) test was used for post hoc multiple comparisons. Dose–response curves were fitted using a four-parameter logistic (4PL) model to estimate EC_50_ values for L-arginine concentrations. All experiments were conducted with five biological replicates and three technical replicates per treatment. For statistical testing, the average of the technical replicates was used per biological replicate. Statistical significance was set at *p* < 0.05.

## 3. Results

### 3.1. Soil Characteristics and Preliminary Isolates

The sampled soils were acidic (pH~3.6), with high aluminum saturation and organic matter content, typical of highly weathered Ultisols. Nitrogen levels were relatively low, with a C:N ratio of 24.3, while nitrate and ammonium concentrations were marginal. Iron was predominantly present in oxide forms. These chemical characteristics were consistent with low-inorganic-N environments where facultative bacteria may rely on organic nitrogen sources (Table 1). 16S rRNA gene sequencing identified high identity scores (>98%) for AC1, AC2, and AC5 (Table 2), confirming their close affiliation with known genera (*Bacillus*, *Collimonas*, and *Paraburkholderia*, respectively). Strain AC7 initially matched *Paenibacillus borealis* at 97.98%, below the usual 98% threshold. Only strain AC7 yielded a band positive for *bnos*-specific PCR. Whole-genome sequencing was conducted to confirm its taxonomic status.

### 3.2. bnos Amplification and Phylogenetic Placement

**Figure 1:**
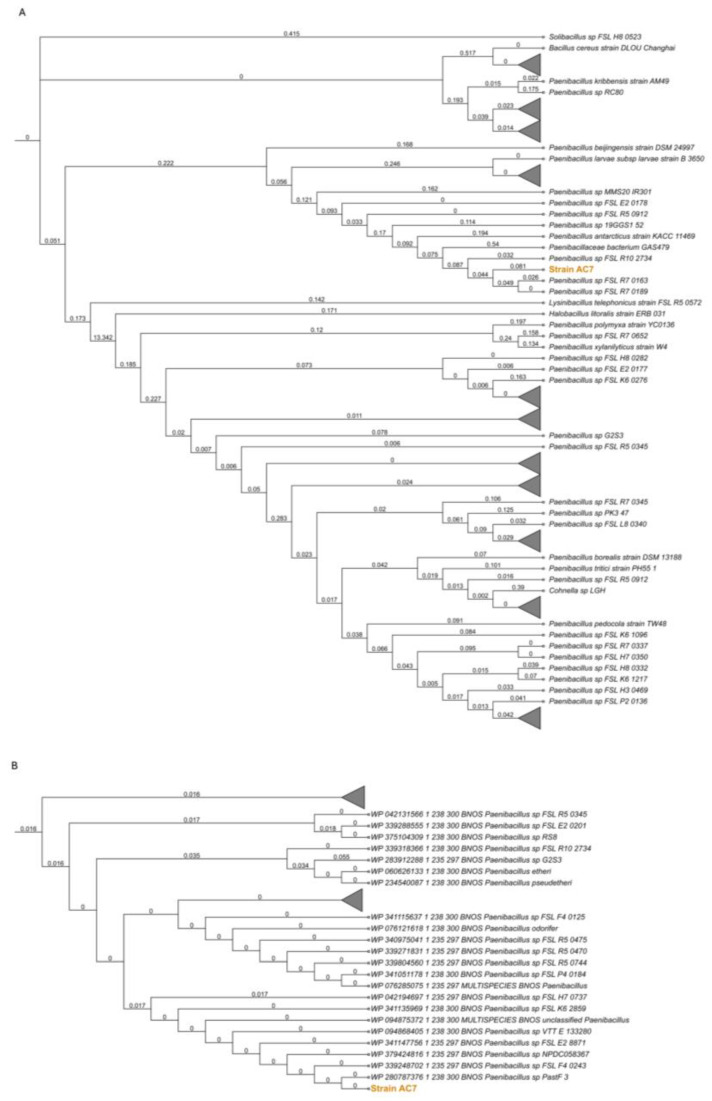
Phylogenetic analysis of the *bnos* in the strain AC7. (**A**) Maximum likelihood tree of the amplified *bnos* gene nucleotide sequences from strain AC7 and related strains. The branch lengths indicate evolutionary distances. Collapsed nodes (triangles) correspond to clades with an average branch length ≤ 0.05. (**B**) The phylogenetic tree is based on the translated bNOS amino acid sequence of strain AC7 compared to homologous entries retrieved from the databases. Collapsed nodes represent clades containing more than 37 sequences.

The amplified *bnos* sequence showed 98.95% identity (E-value 3e^−89^) with the closest sequence in GenBank. A nucleotide-based phylogenetic tree (Figure 1A) positioned the *bnos* sequence within a cluster of *Paenibacillus* strains lacking specific species assignments but closely related to the reported isolates identified through whole-genome sequencing. Translation of the nucleotide sequence into amino acids revealed a 57 amino acid fragment within a 370 amino acid region, showing high similarity to nitric oxide synthase oxygenase (NOS-ox). Protein BLAST analysis revealed a significant match (e-value: 7e^−31^) with sequences specific to the genus *Paenibacillus*. An amino-acid-based tree (Figure 1B) confirmed similar grouping at 98.4% identity, suggesting that the strain AC7 belongs to the *Paenibacillus* lineage, supported by the bootstrap values obtained.

### 3.3. Phylogenetic Analysis and Biochemical Characterization

**Figure 2:**
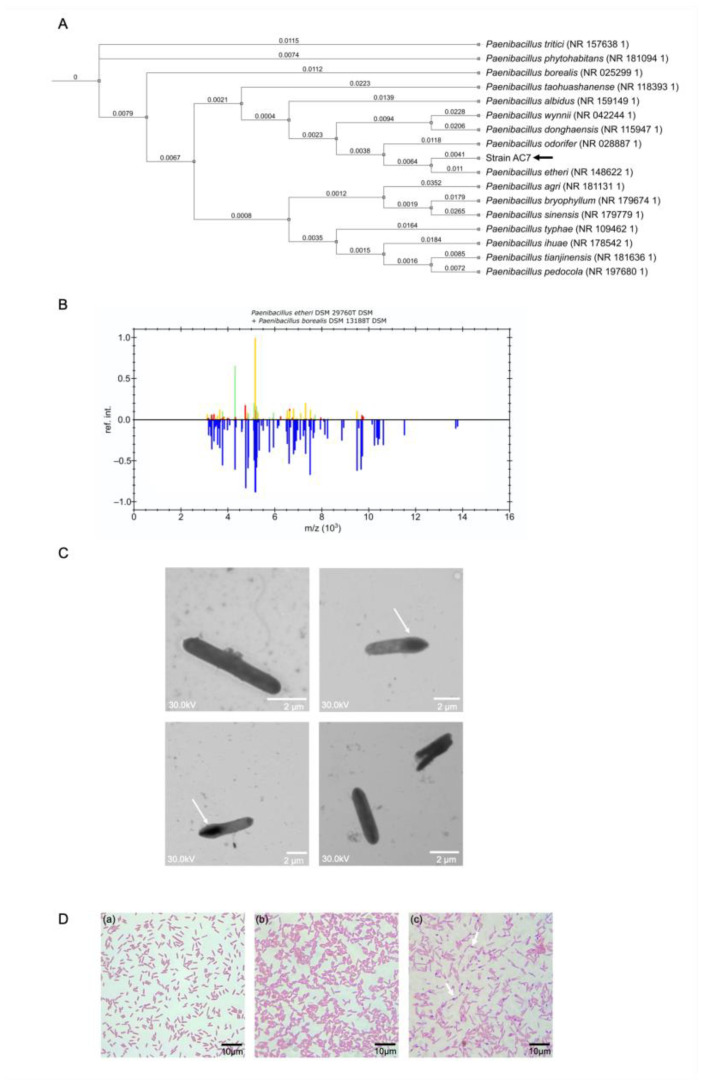
Phylogenetic and morphological characterization of strain AC7 (black arrow). (**A**) Maximum likelihood phylogenetic tree based on the 16S rRNA gene sequences. (**B**) MALDI-TOF MS referenced intensity pattern (ref.int) of strain AC7 (blue) compared with *Paenibacillus etheri* DSM 29760T (yellow) and *P. borealis* DSM 13188T (green), as a function of mass-to-charge ratio (*m*/z). Red bars indicate matching peaks shared between AC7 and the reference strains. Limited spectral overlap supported its distinction as a novel species. (**C**) HV-STEM micrographs of strain AC7. Rod-shaped morphology with clear terminal endospores (white arrows). (**D**) Microscopic observations of strain AC7; (a) Gram staining; (b) endospore staining (malachite green/safranin) of 12 h culture with no visible spores. (c) Endospore staining of 72 h culture revealed terminal spores (white arrows).

The 16S rRNA phylogenetic tree (Figure 2A) positioned the strain AC7 within the genus *Paenibacillus*, forming a distinct clade closely related to *Paenibacillus etheri*, with an identity value of 97.98% (Table 2). Scanning electron microscopy revealed rod-shaped cells (2.4 ± 1.2 µm × 0.6 ± 0.2 µm) with smooth surfaces (Figure 2B). The MALDI-TOF MS profile did not match any known *Paenibacillus* entry, suggesting the presence of a novel taxon (Figure 2C). The strain AC7 displayed as Gram-positive (Figure 2D) and produced endospores (Figure 2D. The biochemical and physiological features of strain AC7 were compared with those of its closest phylogenetic relatives (*Paenibacillus etheri* and *P. borealis*) as shown in Table 3. The differences observed in oxidase activity, carbohydrate utilization, and growth parameters (pH and temperature range) support the delineation of strain AC7 as a novel species within the genus *Paenibacillus*.

### 3.4. Utilization of Carbon and Nitrogen Substrates

**Figure 3:**
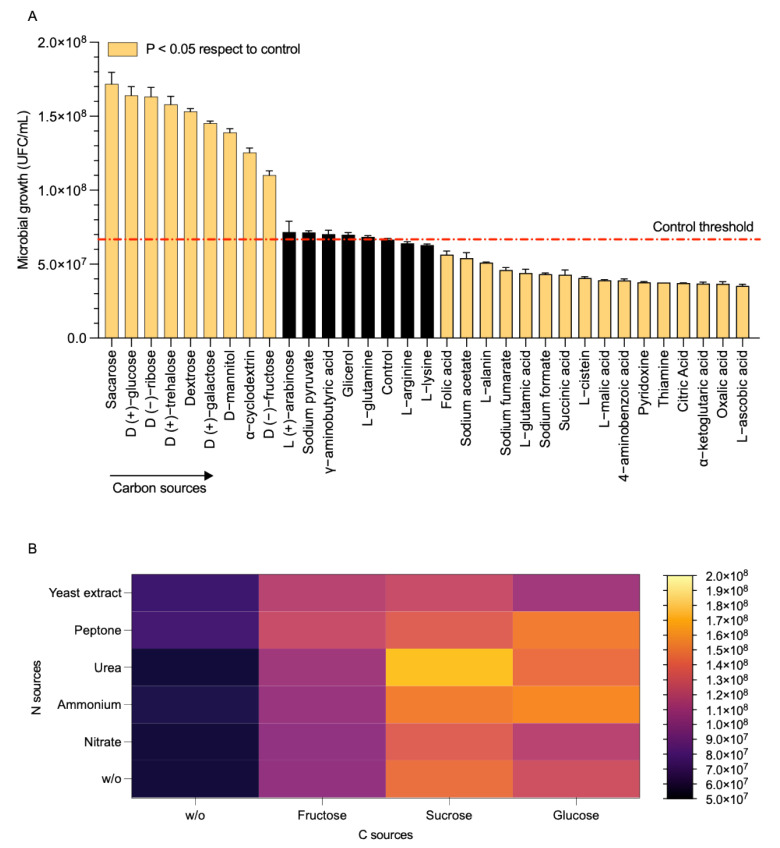
Utilization of carbon and nitrogen sources by *Paenibacillus* strain AC7. (**A**) Growth (CFU/mL) in various carbon sources for 48 h at 30 °C. The dashed red line indicates the control threshold. Carbon sources that significantly reduced or increased growth (*p* < 0.05) are shown in orange; black bars indicate no significant differences. (**B**) Heatmap of CFU/mL under different nitrogen–carbon combinations, with deeper color intensity signifying higher cell counts. Data represent mean values of biological triplicates. W/o: without treatment. N = 5.

Strain AC7 reached approximately 1.7 × 10^8^ CFU/mL when fed with sucrose or glucose whereas it achieved ~4.0–5.0 × 10^7^ CFU/mL with organic acids such as L-ascorbic acid and oxalic acid (Figure 3A, Table 3). Colonies developed in nitrogen-free medium at 48 h, suggesting a free-living nitrogen-fixation mechanism (Table 3). Urea combined with sucrose and ammonium combined with glucose had the highest yields, exceeding 1.5 × 10^8^ CFU/mL, while nitrate with fructose remained below 1.3 × 10^8^ CFU/mL (Figure 3B). These findings confirm strong preference for simple carbohydrates and the versatile use of nitrogen sources (Figure 3B; Table 3).

### 3.5. Bacterial NO Production

**Figure 4:**
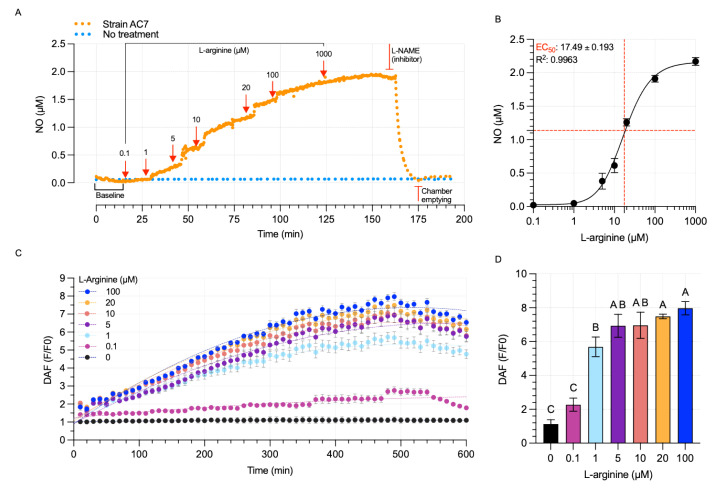
Nitric oxide production in strain AC7. (**A**) NO levels measured by an amperometric electrode in response to 0–1000 µM L-arginine. Red arrows indicate the time points of L-arginine addition; L-NAME (inhibitor) and chamber emptying are also marked in red. Strain AC7 (orange), no treatment control (blue). (**B**) Non-linear dose–response fitted using the highest NO concentrations recorded. The plot shows EC50 values; dashed lines indicate the EC_50_ concentration (vertical) and corresponding NO level (horizontal). (**C**) Time-resolved DAF-FM fluorescence (F/F₀) in cultures treated with increasing concentrations of L-arginine (0–100 µM). Lines connect mean values; error bars indicate standard deviations. N = 5. (**D**) Time-resolved fluorescence (F/F₀) at the end of incubation time. Uppercase letters indicate significant differences at *p* < 0.05. N = 5.

Strain AC7 showed a concentration-dependent increase in extracellular NO when exposed to 0.1–1000 µM L-arginine. NO levels were observed from 0.08 µM at 0.1 µM L-arginine to 1.94 µM at 1000 µM; however, no further rate increase was observed above 100 µM (Figure 4A). A bNOS inhibitor (L-NAME) drastically reduced NO levels, confirming that the enzymatic synthesis of NO is dependent on nitric oxide synthase (Figure 4B). The EC50 for L-arginine was 17.49 µM (R^2^: 0.996) (Figure 4B). Intracellular NO measurements with DAF-FM indicated higher intensities at 1–100 µM L-arginine, with no further gain beyond 100 µM (Figure 4C,D). Cultures deficient in L-arginine showed no detectable NO levels confirming the substrate-dependent nature of NO production (Figure 4D).

### 3.6. Genome Characterization and Phylogenetic Studies

**Figure 5:**
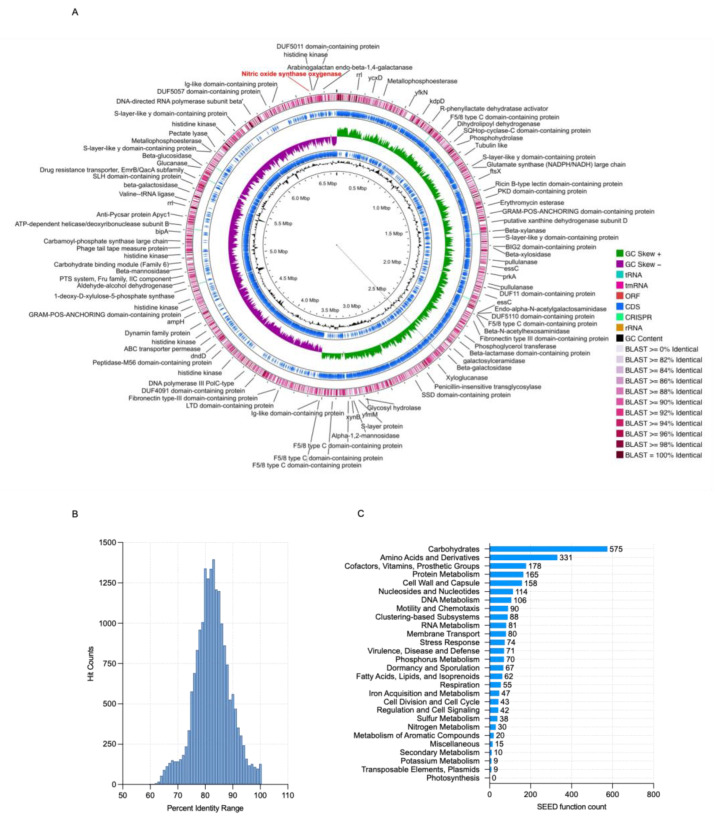
Genome map and predicted features of *Paenibacillus* strain AC7. (**A**) Circular genome showing, from outer to inner rings, BLAST identity (%), rRNA genes, putative CRISPRs, coding sequences (CDS) on the forward strand, GC skew (+/–), CDS on the reverse strand, and GC content. The red line marks the region encoding nitric oxide synthase (bNOS) between 6,594,645 bp and 6,595,766 bp. (**B**) BLAST-based percent-identity distribution compared to *Paenibacillus etheri* (LCZJ02000018.1). (**C**) Functional annotation groups the annotated genes into key categories such as metabolism, transport, stress response, signaling, and antibiotic resistance.

Through whole-genome sequencing and the hybrid assembly of *Paenibacillus* sp. strain AC7 (BioSample SAMN47264457), we obtained a complete genome of 6.79 Mb in length, with a GC content of 43.8% and an estimated coverage of 147.8× (Table 4). The assembly consisted of a single contig and showed no ambiguous bases or misassemblies. Genome completeness and contamination, assessed by BUSCO and CheckM, were 99.8% and 0.9%, respectively. No plasmids were identified (Figure 5A). A total of 6080 coding sequences (CDS) were predicted, along with 10 copies of each ribosomal RNA gene (5S, 16S, and 23S), 93 tRNA genes, and one tmRNA.

To confirm taxonomic identity, we performed a comparative genomic analysis against *Paenibacillus etheri* (RefSeq: GCF_001012825.1). The alignment revealed 577 to 19,391 features with sequence identity >96%, including 121 regions with 100% identity (Figure 5B). Functional annotation (Figure 5C) identified 30 nitrogen-associated genes, including one nitric oxide synthase, twelve genes for ammonia assimilation, four for denitrification, three for nitrate/nitrite ammonification, one dissimilatory nitrite reductase, seven for allantoin utilization, and two for cyanate hydrolysis. Additionally, five multilocus sequence typing (MLST) markers were detected: glycerol kinase, guanylate kinase, triosephosphate isomerase, and two acetyl-CoA acetyltransferases.

**Figure 6:**
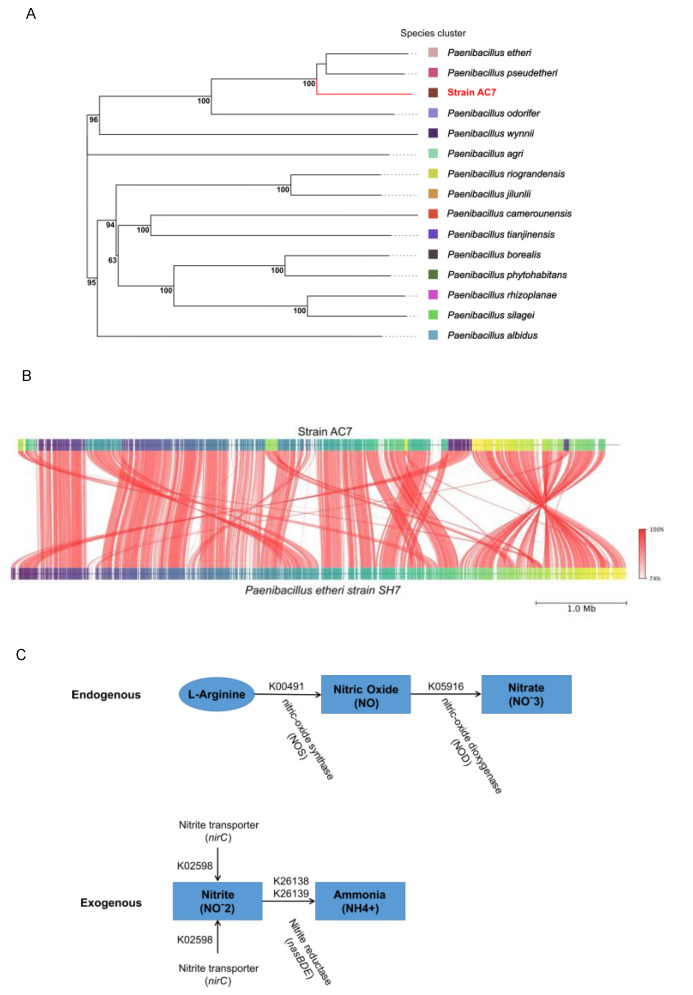
Whole-genome evolutionary relationship of strain AC7. (**A**) dDDH-based tree illustrating the evolutionary placement of the strain AC7 among *Paenibacillus* genus. The color-coded squares represent separate *Paenibacillus* clusters. Support values at the nodes reflect the confidence of each branch based on dDDH analysis. (**B**) Synteny plot comparing the genome of strain AC7 with the reference genome of *P. etheri* SH7. Red lines indicate conserved collinear regions with high sequence identity, while the intensity of the red color corresponds to the degree of nucleotide similarity, as shown in the scale bar. Genome coordinates are displayed on the horizontal axis. (**C**) Schematic of L-arginine metabolic routes in AC7. Ovals depict initial substrates and rectangles show final products. Solid arrows mark enzymatic steps, with KEGG codes above and enzyme names below. The endogenous pathway uses nitric oxide synthase (K00491) to convert L-arginine into NO under aerobic conditions while the exogenous pathway processes nitrite and nitrate.

The dDDH analysis (Figure 6A, Table S3) supported the phylogenetic distinction of strain AC7 as a novel species. AC7 clustered with *P. etheri* (64.9%) and *P. pseudetheri* (61.3%) while showing less similarity with *P. odorifer* (52.7%) and distant values with *P. tianjinensis* (17.1%) and *P. wynnii* (16.7%). The G+C content differences also supported this: 0.20% with *P. etheri*, 0.29% with *P. pseudetheri*, and 5.77% with *P. tianjinensis*. FastANI analysis confirmed the closest match to *P. etheri* with 94.75% identity and 89.6% MSA AA, as shown as a synteny plot in Figure 6B. Genome-based metabolic reconstruction revealed two potential NO-related pathways in *Paenibacillus nitricinens* strain AC7: an endogenous aerobic route, involving nitric oxide synthase (*bNOS*, K00491) and nitric oxide dioxygenase (*NOD*, K05916), and an exogenous route (not complete) dependent on nitrite uptake (*nirC*, K02598) and reduction via nitrite reductase (*nasBDE*; K26138, K26139) (Figure 6C). The complete pathways observed in the flux balance analysis (FBA) are shown in Figure S3. Also, these enzymatic steps are not explicitly represented in the curated metabolic model used for flux balance analysis (FBA). Despite this, the nitrogen pathways obtained from FBA revealed high fluxes through glycolysis, pyruvate conversion, and NAD(P)H-generating steps, suggesting a strongly oxidative metabolic context. This profile supports the potential for sustained aerobic NO production via bNOS, even in the absence of canonical denitrification or nitrification routes (Figure S3).

## 4. Discussion

### 4.1. Aerobic NO Production via bNOS in Paenibacillus Nitricinens sp. nov.

This paper provides the first direct evidence of L-arginine-dependent nitric oxide synthase (bNOS)-mediated NO production under aerobic conditions in a free-living *Paenibacillus* species. While nitric oxide synthase activity has been characterized in *Bacillus subtilis* [10], and predicted in other genera [6,7], functional validation in native, non-symbiotic soil strains has remained elusive [2,3,19]. Here, strain AC7, now proposed as *Paenibacillus nitricinens* sp. nov., harbors a *bnos*-like gene confirmed by PCR (Figure 1), whole-genome sequencing (~6.79 Mb; Figure 5 and Figure 6), and phylogenomic comparison (Figure 1B), aligning with previous observations that some soil bacteria harbor cryptic NO biosynthesis mechanisms [17].

Dose–response assays showed that NO accumulates significantly at L-arginine concentrations as low as 0.1 µM, with saturation observed above 100 µM (Figure 4A). The estimated EC_50_ of 17.49 µM (Figure 4B) indicates high substrate affinity, consistent with enzymatic NO production. Inhibition with L-NAME, a specific NOS inhibitor [41,42], and the absence of NO production in L-arginine-free controls reinforce the enzymatic and substrate-dependent nature of this process. These results are consistent with bNOS activity previously described in *Bacillus subtilis* [10] and other Gram-positive bacteria [43,44] but provide the first in vivo evidence in a wild-type *Paenibacillus*.

Whole-genome annotation revealed no canonical denitrification genes (e.g., *narG-H-I-J*, *napA-B*, *nirK/S*, *norB*, or *nosZ*) (Table 4), indicating that NO synthesis in AC7 is independent from classical denitrification pathways, which typically require oxygen limitation and nitrogen oxyanions [4,5]. Additionally, the genome encodes a nitric oxide dioxygenase (NOD; K05916), potentially forming an NO production–detoxification loop that mitigates toxicity while enabling redox signaling under aerobic conditions (Figure 6C) [45]. The strain’s *bnos* sequence exhibits notable divergence compared to other *Paenibacillus* strains (Figure 1), suggesting evolutionary specialization [46]. The combination of gene presence, dose-dependent NO production, high affinity for L-arginine, and inhibitor sensitivity collectively support bNOS as the primary route for NO synthesis in *P. nitricinens*.

### 4.2. Taxonomic Distinction and Functional Characterization

Strain AC7 meets multiple genomic and phenotypic criteria supporting its status as a novel species. Its 16S rRNA identity with *P. borealis* is 97.98%, below the 98.7% species threshold [47], while dDDH (64.9%) and ANI (94.75%) values also fall below accepted cutoffs for species-level distinction (Table S3) [13]. Whole-genome alignment and synteny plots (Figure 6B) confirmed genomic divergence, and MALDI-TOF MS profiling revealed no match with any existing *Paenibacillus* reference (Figure 2B). In addition to genomic distinctiveness, AC7 presents unique physiological and biochemical characteristics (Table 3). It is a Gram-negative, rod-shaped, endospore-forming facultative anaerobe (Figure 2C,D), with a growth temperature range of 12–30 °C and tolerance to acidic conditions (pH 6.0–8.5), which matches its native soil environment (Table 1). Biochemically, AC7 is oxidase-negative and catalase-positive and differs from closely related species in lactose fermentation and citrate utilization. These phenotypic traits further support species delineation and ecological specialization [11,12,13].

### 4.3. Metabolic Versatility and Environmental Adaptation

*P. nitricinens* displays broad metabolic flexibility as demonstrated by its robust growth on various carbon and nitrogen sources (Figure 3A,B). It reached maximal cell density on glucose and sucrose and could grow on amino acids and organic acids such as oxalate and ascorbate, which are common in forest soils [48]. The strain also grew on nitrogen-free media, indicating potential nitrogen fixation capacity, consistent with previous observations in the genus [13]. Genomic analysis identified genes associated with nitrogen assimilation and detoxification including hydroxylamine reductase, glutamine synthetase, and cyanate lyase, supporting this interpretation (Figure 6; Table 4) [49,50]. Flux balance analysis (Figure S3) revealed strong metabolic fluxes through glycolysis, the TCA cycle, and amino acid conversion pathways, which could support sustained NO production from L-arginine under aerobic conditions [51]. This metabolic profile suggests that AC7 may occupy a niche where amino acid catabolism and redox balance are critical, particularly in organic-rich but nitrogen-poor soils such as those in southern Chile [1,19,24].

### 4.4. Implications for Soil Nitrogen Cycling

These findings challenge the traditional view that NO emissions in soils are primarily associated with anaerobic denitrification [1,5]. Instead, *P. nitricinens* demonstrates an alternative, aerobic route of NO production mediated by bNOS and independent of nitrate or nitrite availability. This pathway could contribute to NO fluxes in well-aerated soils and affect downstream microbial processes such as nitrification inhibition or biofilm signaling [3,42]. The strain’s ability to generate NO in response to micromolar L-arginine concentrations, in the absence of oxygen limitation, introduces a new ecological dimension to microbial nitrogen cycling. The co-occurrence of bNOS and NOD may allow controlled NO turnover without causing cellular damage, supporting its possible role in signaling rather than nitrogen oxide respiration [42,52].

Soils with fluctuating redox conditions, such as those found in temperate rainforests, frequently harbor facultative bacteria capable of integrating both aerobic and anaerobic metabolisms [17,18]. In such environments, *P. nitricinens* could serve as a cryptic but significant source of NO, influencing microbial community structure and nitrogen retention. Its discovery provides a foundation for further studies into non-canonical nitrogen transformations, especially in pristine ecosystems with minimal nitrogen deposition [53].

### 4.5. Potential Regulation of bNOS and Future Directions

Although this study confirmed functional nitric oxide production via bNOS under aerobic, L-arginine-supplemented conditions, the regulatory mechanisms governing *bNOS* expression in *P. nitricinens* remain unknown. In other bacteria such as *Bacillus subtilis*, bNOS expression is linked to oxidative stress and iron homeostasis, often modulated by transcription factors such as PerR and Fur [54]. These regulatory pathways allow cells to fine-tune NO synthesis in response to redox status or nutrient limitation. Given the presence of multiple nitrogen metabolism genes and the oxidative metabolic context inferred from flux balance analysis (Figure S3), it is plausible that bNOS expression *in P. nitricinens* may also respond to environmental cues such as oxygen concentration, the carbon/nitrogen ratio, or the availability of metal cofactors like iron or tetrahydrobiopterin analogues [53,55,56]. The co-localization or genomic proximity of *bNOS* to stress-response- or redox-related genes could further support this model but requires transcriptomic and proteomic validation.

Future studies could explore *bNOS* gene regulation through transcriptome profiling under varying substrate and oxygen conditions or using promoter–reporter constructs. Additionally, the potential for post-translational regulation (e.g., via flavoprotein interactions or NO feedback inhibition) remains an open and promising line of investigation. These efforts would not only clarify how bNOS activity is controlled in environmental *Paenibacillus* species but also help assess its ecological role in fluctuating soil environments.

## 5. Conclusions

*Paenibacillus nitricinens* is a novel species capable of generating nitric oxide (NO) under aerobic conditions through the L-arginine-dependent nitric oxide synthase (bNOS) pathway. Genome sequencing and phylogenomic analyses confirmed their distinction from closely related *Paenibacillus* taxa while dose–response experiments showed NO production even at low micromolar concentrations of L-arginine. The inhibitory effect of L-NAME supports bNOS-mediated catalysis, suggesting that alternative mechanisms of aerobic NO formation may occur in more bacterial taxa than previously recognized, warranting a broader investigation. In organic-rich oxygenated soils, such a mechanism could expand the known pathways of nitrogen cycling by releasing NO without relying on denitrification. The presence of multiple nitrogen metabolism genes in *P. nitricinens* further underscores its metabolic flexibility under dynamic environments. These results highlight the putative bNOS-dependent NO synthesis pathway as a potential candidate mechanism for aerobic NO production. Further research is required to validate the functional role of *Paenibacillus* and related genera in local and global nitrogen fluxes.

## Figures and Tables

**Table 1:** Chemical soil characteristics of sampling location.

	Parameter	Units	Value
Site characteristics	Coordinates	–	40°12′ S–73°26′ W
	Parent material	–	Metamorphic, mica schist
	Soil order ^1^	–	Ultisol
	Clay type ^2^	–	Q, I, K
	Texture ^3^	–	CL
	MAT ^4^	°C	9.5
	MAP ^5^	mm a^−1^	4000
	Elevation	m.a.s.l.	1048
	Vegetation ^6^	–	DW, LP; NN, NP, PN, SC
Soil chemical properties	Total C	%	9.2 ± 2.1
	Total N	%	0.47 ± 0.01
	C:N ratio	–	24.3
	NO_3_^−^	%	0.3 ± 0.0
	NH_4_⁺	%	0.16 ± 0.02
	pH (water)	–	3.9 ± 0.2
	Al saturation	%	80 ± 9.2
	Total Fe	g kg^−1^ soil	10.4 ± 0.3
	Fe^2^⁺	g kg^−1^ soil	5.3 ± 0.6
	Fe^3^⁺	g kg^−1^ soil	5.1 ± 0.3

^1^ Soil order [25]. ^2^ K: kaolinite, Q:quartz, I: illite, [26]. ^3^ Silty loam, CL: clay loam [26]. ^4^ MAT: Mean annual temperature. ^5^ MAP: Mean annual precipitation. ^6^ vegetation: DW: *Drimys winteri*; LP: *Laureliopsis philippiana* (Looser) Schodde (Monimiaceae); NN: *Nothofagus nitida* (Phil); NP: *Nothofagus pumulio*; PN: *Podocarpus nubigena* Lindl; SC: *Saxegothaea conspicua* (Lindl.).

**Table 2:** Taxonomic classification of bacterial isolates identified by 16S rRNA sequencing and *bnos*-specific PCR screening.

Strain	Closest Accession	Closest Organism	Identity (%)	Coverage (%)	E-Value	Length (bp)	*bnos* -PCR	Accession Number
AC1	NR_104919.1	*Bacillus tequilensis*	99.86	100	0.0	1531	-	PV535646
AC2	NR_179598.1	*Collimonas antrihumi*	99.31	100	0.0	1425	-	PV529833
AC5	NR_074325.2	*Paraburkholderia bryophila*	98.98	100	0.0	1484	-	PV535647
AC7	NR_025299.1	*Paenibacillus borealis*	97.98	100	0.0	1540	+	PV248128

**Table 3:** Comparison of biochemical and physiological characteristics of strain AC7 with closely related *Paenibacillus* species based on 16S rRNA gene similarit; *Paenibacillus etheri* (BacDive 132397), and *Paenibacillus odorifer* (BacDive 11560).

	Characteristics	Strain AC7	*P. etheri*	*P. odorifer*
Morphological characteristics	Gram Stain	-	+	-
	Cell shape	Rod-shaped	Rod-shaped	Rod-shaped
	Spore formation	+	+	+
	Motility	-	+	+
Physiological traits	Oxygen tolerance	Facultative anaerobe	Facultative anaerobe	Facultative anaerobe
	pH range	6.0–8.5	7.0–8.0	6.0–8.0
	Temperature range (°C)	12–30	5–35	5–35
Biochemical tests and enzymatic activity	Catalase	+	+	+
	Oxidase	-	-	-
	Citrate	-	-	+
	Urease	-	-	-
	Starch utilization	+	+	+
	Indole	-	-	-
	Voges–Proskauer	+	+	+
	Methyl Red	-	-	-
Fermentation	Glucose	+	+	+
	Lactose	-	-	+
	Mannitol	+	+	+
Functional capabilities	P solubilization	-	ND	ND
	Free-nitrogen culture	+	ND	ND
Genomic information	DNA G+C content	43.79%	44.30%	44.35%

“+”: positive; “-“: negative; ND: not determined.

**Table 4:** Genome assembly and annotation summary for *Paenibacillus* sp. strain AC7.

Category	Feature	Value
Annotation and gene prediction	Genome size	6,790,125 bp
	Number of contigs	1
	Largest contig	6,790,125 bp
	N50/L50	6,790,125 bp/1
	Genome topology	Putative circular
	GC content	43.80%
	Sequencing coverage	147.8×
	Ambiguous bases (Ns)	0
	Misassemblies	0
	Mismatches/100 kbp	7697.61
	Indels/100 kbp	104
	CDS (protein-coding genes)	6080 (Prokka annotation)
	rRNA genes	30 (10 × 5S, 10 × 16S, 10 × 23S)
	tRNA genes	93
	tmRNA gene	1
Completeness metrics	BUSCO completeness (bacillales_odb10)	99.8% (98.9% single-copy, 0.9% duplicated)
	Fragmented BUSCOs	0.20%
	Missing BUSCOs	0%
Comparative alignment	Genome fraction aligned to reference	41.80% (*Paenibacillus etheri*)
	100% identity hits	121
	Hit range with >96% identity	577–19,391
Other genomic features	Putative plasmids	None detected (PlasFlow classification)
	MLST markers	5 (glpK, gmk, tpiA, and 2× acat)
	Biosynthetic gene clusters	4 (3 RiPPs, 1 terpene; antiSMASH v6.1.1)
	Antimicrobial resistance genes	None detected (staramr analysis)
Submission details	BioProject ID	PRJNA1233252
	BioSample ID	SAMN47264457
	Taxonomy ID	3367691

## Data Availability

The whole-genome sequence of the *Paenibacillus nitricinens* strain AC7 has been deposited in GenBank under BioProject accession number PRJNA1233252 (SRA: SRX26308475 and SRX26308474). The 16S rRNA gene sequence is available in GenBank with the accession number provided in Table 2. The raw MALDI-TOF/TOF MS spectrum data are available at Zenodo under the following DOI: 10.5281/zenodo.15298880. All other relevant data supporting the findings of this study are available from the corresponding author upon reasonable request. The type strain has been deposited in the Colección Chilena de Cultivos Tipo (CCCT, WDCM 1111) under accession number CCCT 25.01.

## Supplementary Materials

The following supporting information can be downloaded at: https://www.mdpi.com/article/10.3390/biology14060733/s1, Method S1: Design of *bnos* primers and PCR; Figure S1: Phylogenetic analysis of bacterial nitric oxide synthase (*bnos*) genes; Figure S2: Phylogenetic similarity of bacterial nitric oxide synthase (*bnos*) genes in selected genus; Table S1: Organisms used as reference for *bnos* primer design; Table S2: Organisms used as reference for *bnos* primer design; Table S3: Pairwise dDDH values between the strain AC7 and TYGS genomes; Figure S3: Nitrogen Metabolism pathway in strain AC7.

## Abbreviations

The following abbreviations have been used in this manuscript:

ANI	Average nucleotide identity
CCCT	Colección Chilena de Cultivos Tipo
CFU	Colony-forming unit
dDDH	Digital DNA–DNA hybridization
FASTME	Fast Minimum Evolution
FBA	Flux balance analysis
GBDP	Genome BLAST Distance Phylogeny
GC skew	Guanine–cytosine skew
HSD	Tukey’s honest significant difference
MSA AA	Multiple-sequence alignment (amino acid)
OAT	OrthoANI Tool
OD600	Optical density at 600 nanometers
PBS	Phosphate-buffered saline
pHMM	Profile hidden Markov model
rpm	Revolutions per minute
SNAP	S-nitroso-N-acetylpenicillamine
SRA	Sequence Read Archive
tmRNA	Transfer–messenger RNA
TYGS	Type (Strain) Genome Server
WGS	Whole-genome sequencing
