# Supplementary material for: Functional and Genomic Evidence of L-Arginine-Dependent Bacterial Nitric Oxide Synthase Activity in Paenibacillus nitricinens sp. nov
Source: Biology (Basel). 2025 Jun 19;14(6):733. doi: 10.3390/biology14060733 (PMC12189478; doi:10.3390/biology14060733)
Supplement: Supplementary file 1 [file biology-14-00733-s001.zip › biology-3672171-supplementary.pdf]

Supplementary Methodology S1

Method S1: Design of bnos primers and PCR.

Primer design and PCR validation were performed to detect the presence of the *bnos* gene in the isolated strains, facilitating the selection of relevant samples for in vitro studies. A comprehensive search was conducted in the GenBank (NCBI) database using the keywords "Nitric oxide synthase" and "Bacteria." All retrieved gene sequences (Table S1) were analyzed using the NGphylogeny.fr platform with the advanced method, which included MAFFT for multiple sequence alignment, BMGE for removing poorly aligned regions, and PhyML for phylogenetic inference based on the maximum-likelihood method to construct the tree (Figure S1). Based on the alignment, were selected 13 *Bacillus* and 11 *Paenibacillus* species exhibited the highest genus-level convergence for the *bnos* gene on sequence homology (Figure S2, and Table S2). Strains AC2 (*Collimonas antrihumi*) and AC5 (*Paraburkholderia bryophila*) were removed from the analysis, as no *bnos* genes sequences were found for their respective genera in GenBank. A single consensus sequence was obtained, representing conserved regions with high homology across all analyzed sequences. Primers were designed based on three criteria: 1. The amplified fragment size was restricted to 100–250 bp. 2. The last three nucleotides at the 3′-end of both forward and reverse primers had to be identical across all 24 sequences, and 3. No more than one nucleotide mismatch was allowed, with "low homology" defined as positions where fewer than 18 of the 24 sequences shared the same nucleotide. The primers were validated using Primer-BLAST, and AmplifX software to detect %GC content and potential secondary structures. Two primer oligonucleotides were synthesized, bNOSPF1: 5′- CCC TTT AAC GGC TGG TAT ATG G -3′, and bNOSPR1: 5′- TAT GAT GGT CAA CGA TGC TGA C -3′. The primer pair was predicted to amplify a 211 bp fragment.

Under experimental conditions, the *bnos* gene PCR was performed using SapphireAmp® Fast PCR Master Mix (Takara Bio, RR350A) in 50 μL per reaction using the gDNA from isolated strains as template. The thermal cycling conditions were as follows: initial denaturation at 98°C for 2 minutes, followed by 35 cycles of denaturation at 94°C for 15 seconds, annealing at an optimized temperature (57°C) for 15 seconds, and extension at 72°C for 20 seconds, and a final extension was conducted at 72°C for 2 minutes. PCR products within the 100–250 bp range were considered positive. Only strain AC7 was successfully amplified with the designed primers. The PCR product from strain AC7 was purified and sequenced using the Sanger method on an ABI 3500 Genetic Analyzer to confirm its identity. The obtained nucleotide sequences were analyzed using BLAST (blastn and blastx), both with and without restriction to *Bacillus* and *Paenibacillus* genera, to identify homologous sequences for nucleotide and protein sequences, confirming the specificity of the amplified fragments.

Supplementary Table S1

Table S1. Organisms used as reference for *bnos* primer design.

tax_id	Taxonomy name	Symbol	GeneID	other_designations
235986	*Actinacidiphila glaucinigra*	OIE69_RS07410	95784651	nitric oxide synthase oxygenase
713604	*Amycolatopsis mediterranei S699*	RAM_RS20880	92871887	nitric oxide synthase oxygenase
491915	*Anoxybacillus flavithermus WK1*	AFLV_RS01795	7036530	nitric oxide synthase oxygenase
2026189	*Bacillus albus*	QRY64_RS30030	83639132	nitric oxide synthase oxygenase
293387	*Bacillus altitudinis*	ID12_RS08500	66362311	nitric oxide synthase oxygenase
1390	*Bacillus amyloliquefaciens*	nosA	75094477	nitric oxide synthase
261594	*Bacillus anthracis str. 'Ames Ancestor'*	GBAA_RS27735	45025270	nitric oxide synthase oxygenase
1452	*Bacillus atrophaeus*	nosA	92915959	nitric oxide synthase
1455	*Bacillus badius*	B1A98_RS14315	92777922	nitric oxide synthase oxygenase
1396	*Bacillus cereus*	FORC47_RS28110	72452093	nitric oxide synthase oxygenase
315749	*Bacillus cytotoxicus NVH 391-98*	BCER98_RS19935	33899190	nitric oxide synthase oxygenase
1664069	*Bacillus glycinifermentans*	EQZ20_RS04430	82851926	nitric oxide synthase oxygenase
260554	*Bacillus halotolerans*	nosA	50134445	nitric oxide synthase
1925021	*Bacillus haynesii*	H2R00_RS11100	76974319	nitric oxide synthase oxygenase
483913	*Bacillus inaquosorum*	nosA	76977197	nitric oxide synthase
324767	*Bacillus infantis*	ABXS71_RS18330	97351203	nitric oxide synthase oxygenase
1402	*Bacillus licheniformis*	SR852_RS21030	92862633	nitric oxide synthase oxygenase
2026190	*Bacillus mobilis*	BAQ44_RS25485	92803288	nitric oxide synthase oxygenase
72360	*Bacillus mojavensis*	nosA	76981495	nitric oxide synthase
1405	*Bacillus mycoides*	EXW63_RS14325	66265008	nitric oxide synthase oxygenase
2026193	*Bacillus nitratireducens*	Q3V31_RS19180	92885979	nitric oxide synthase oxygenase
2026187	*Bacillus pacificus*	LMD38_RS26605	69534537	nitric oxide synthase oxygenase
1648923	*Bacillus paralicheniformis*	CP943_RS04435	56670525	nitric oxide synthase oxygenase

2026194	*Bacillus paramycoides*	BAU28_RS19335	87593508	nitric oxide synthase oxygenase
2026186	*Bacillus paranthracis*	NLJ82_RS26850	75088627	nitric oxide synthase oxygenase
527000	*Bacillus pseudomycoides DSM 12442*	BPMYX0001_RS23800	34215039	nitric oxide synthase oxygenase
315750	*Bacillus pumilus SAFR-032*	BPUM_RS03690	5619959	nitric oxide synthase oxygenase
561879	*Bacillus safensis*	FX981_RS03940	61767581	nitric oxide synthase oxygenase
1177185	*Bacillus siamensis KCTC 13613*	nosA	76426426	nitric oxide synthase
119858	*Bacillus sonorensis*	SK061_RS04705	92851928	nitric oxide synthase oxygenase
1052585	*Bacillus spizizenii TU-B-10*	nosA	11238656	nitric oxide synthase
2054641	*Bacillus stercoris*	nosA	86874775	nitric oxide synthase
224308	*Bacillus subtilis subsp. subtilis str. 168*	nosA	938802	nitric-oxide synthase
1925020	*Bacillus swezeyi*	RBH91_RS04450	92788646	nitric oxide synthase oxygenase
527031	*Bacillus thuringiensis serovar berliner ATCC 10792*	BTHUR0008_RS26075	67469686	nitric oxide synthase oxygenase
155322	*Bacillus toyonensis*	I0K03_RS26475	64186707	nitric oxide synthase oxygenase
2026188	*Bacillus tropicus*	P3F89_RS00250	93005681	nitric oxide synthase oxygenase
72361	*Bacillus vallismortis*	nosA	76985863	nitric oxide synthase
326423	*Bacillus velezensis FZB42*	nosA	93079917	nitric oxide synthase
1890302	*Bacillus wiedmannii*	D4A37_RS28290	51137121	nitric oxide synthase oxygenase
51101	*Brevibacillus agri*	BA6348_RS03810	82809432	nitric oxide synthase oxygenase
45462	*Brevibacillus borstelensis*	V5G20_RS14625	89499866	nitric oxide synthase oxygenase
1393	*Brevibacillus brevis*	EL268_RS31055	61036272	nitric oxide synthase oxygenase
54913	*Brevibacillus formosus*	AA984_RS12260	87585846	nitric oxide synthase oxygenase
54914	*Brevibacillus parabrevis*	IRJ17_RS05500	87610663	nitric oxide synthase oxygenase
2126350	*Brevibacillus porteri*	C7R92_RS20450	95752466	nitric oxide synthase oxygenase
2756	*Brochothrix thermosphacta*	BFC19_RS04730	66536922	nitric oxide synthase oxygenase
243230	*Deinococcus radiodurans R1 = ATCC 13939 = DSM 20539*	nos	69518850	nitric oxide synthase oxygenase
1288484	*Deinococcus wulumuqiensis R12*	nos	59165944	nitric oxide synthase oxygenase

41170	*Exiguobacterium acetylicum*	KKI46_RS02030	88810425	nitric oxide synthase oxygenase
1358421	*Exiguobacterium indicum HHS 31*	N179_RS08850	90837575	nitric oxide synthase oxygenase
307643	*Exiguobacterium profundum*	OE059_RS03540	94371324	nitric oxide synthase oxygenase
1422	*Geobacillus stearothermophilus*	QA416_RS09180	89612948	nitric oxide synthase oxygenase
33941	*Geobacillus thermoleovorans*	GT3570_RS08055	32063572	nitric oxide synthase oxygenase
371036	*Gottfriedia acidiceleris*	B6K90_RS14015	34872364	nitric oxide synthase oxygenase
86665	*Halalkalibacterium halodurans*	FED51_RS04390	87596373	nitric oxide synthase oxygenase
45668	*Halobacillus litoralis*	GLW00_RS18495	78009016	nitric oxide synthase oxygenase
38875	*Heyndrickxia oleronia*	KI370_RS18270	79869368	nitric oxide synthase oxygenase
46224	*Heyndrickxia sporothermodurans*	B5V89_RS16595	62500029	nitric oxide synthase oxygenase
1894	*Kitasatospora aureofaciens*	IE236_RS14125	97485958	nitric oxide synthase oxygenase
67307	*Kitasatospora indigofera*	IE329_RS06325	95351802	nitric oxide synthase oxygenase
67352	*Kitasatospora purpeofusca*	OG715_RS34125	95081000	nitric oxide synthase oxygenase
1552123	*Listeria booriae*	EP57_RS04900	58716760	nitric oxide synthase oxygenase
2115968	*Lysinibacillus capsici*	LCP48_RS01715	74903375	nitric oxide synthase oxygenase
28031	*Lysinibacillus fusiformis*	HR49_RS22605	29442109	nitric oxide synthase oxygenase
1421	*Lysinibacillus sphaericus*	LS41612_RS21725	48278822	nitric oxide synthase oxygenase
582475	*Lysinibacillus xylanilyticus*	ACZ11_RS14065	96599352	nitric oxide synthase oxygenase
582475	*Lysinibacillus xylanilyticus*	ACZ11_RS14165	96599372	nitric oxide synthase oxygenase
1855823	*Macrococcoides canis*	MCCS_RS10185	35296195	nitric oxide synthase oxygenase
69966	*Macrococcoides caseolyticum*	I6G25_RS07260	61129981	nitric oxide synthase oxygenase
150056	*Mammaliicoccus fleurettii*	B2G86_RS11095	86197666	nitric oxide synthase oxygenase
42858	*Mammaliicoccus lentus*	JT690_RS04445	79849439	nitric oxide synthase oxygenase
1296	*Mammaliicoccus sciuri*	DQL71_RS04540	48592317	nitric oxide synthase oxygenase
71237	*Mammaliicoccus vitulinus*	I6J10_RS07325	64116927	nitric oxide synthase oxygenase
1460882	*Microbispora bryophytorum*	ACGFIB_RS03275	97245018	nitric oxide synthase oxygenase

58117	*Microbispora rosea*	ACIBK1_RS29065	97498869	nitric oxide synthase oxygenase
147065	*Microbispora rosea subsp. aerata*	IEY92_RS07720	91536278	nitric oxide synthase oxygenase
47858	*Micromonospora echinofusca*	GA0070610_RS13170	95802471	nitric oxide synthase oxygenase
709883	*Micromonospora zamorensis*	OHQ88_RS33840	91324177	nitric oxide synthase oxygenase
2499688	*Niallia taxi*	QP854_RS19045	87619012	nitric oxide synthase oxygenase
120957	*Nocardia abscessus*	LK455_RS10060	86964053	nitric oxide synthase oxygenase
1824	*Nocardia asteroides*	EL493_RS04790	91514572	nitric oxide synthase oxygenase
1210068	*Nocardia beijingensis NBRC 16342*	NB3_RS10010	96236650	nitric oxide synthase oxygenase
37326	*Nocardia brasiliensis*	CEQ30_RS28375	80365117	nitric oxide synthase oxygenase
134984	*Nocardia fluminea*	AB0F85_RS31480	97473845	nitric oxide synthase oxygenase
1823	*Nocardia otitidiscaviarum*	FOH10_RS11235	80332955	nitric oxide synthase oxygenase
53431	*Nocardia salmonicida*	OHS03_RS29255	91380302	nitric oxide synthase oxygenase
2055889	*Nocardia tengchongensis*	KHQ06_RS36635	95415225	nitric oxide synthase oxygenase
455432	*Nocardia terpenica*	CRH09_RS20790	88359793	nitric oxide synthase oxygenase
65515	*Nonomuraea dietziae*	FHR33_RS37075	95393769	nitric oxide synthase oxygenase
44250	*Paenibacillus alvei*	OB446_RS48080	94490357	nitric oxide synthase oxygenase
1451	*Paenibacillus amylolyticus*	V6668_RS28285	93479456	nitric oxide synthase oxygenase
46240	*Paenibacillus apiarius*	JSQ80_RS26260	77005413	nitric oxide synthase oxygenase
79263	*Paenibacillus chitinolyticus*	PC41400_RS07280	95374629	nitric oxide synthase oxygenase
130049	*Paenibacillus dendritiformis*	L6439_RS13980	73385332	nitric oxide synthase oxygenase
59843	*Paenibacillus glucanolyticus*	WDY75_RS09635	97554306	nitric oxide synthase oxygenase
228574	*Paenibacillus lactis*	J2Z18_RS24525	95406915	nitric oxide synthase oxygenase
147375	*Paenibacillus larvae subsp. larvae*	ERICIV_RS11200	64218999	nitric oxide synthase oxygenase
1349780	*Paenibacillus lautus NBRC 15380*	PLA01S_RS08470	72764898	nitric oxide synthase oxygenase
189426	*Paenibacillus odorifer*	PODO_RS28910	31574286	nitric oxide synthase oxygenase
1087481	*Paenibacillus peoriae KCTC 3763*	KQI_RS0112715	71024572	nitric oxide synthase oxygenase

1036171	*Paenibacillus polymyxa ATCC 842*	CUU60_RS14825	9334853 1	nitric oxide synthase oxygenase
484184	*Paenibacillus taichungensis*	HP548_RS09305	9713089 4	nitric oxide synthase oxygenase
49283	*Paenibacillus thiaminolyticus*	FLT43_RS02790	7699490 7	nitric oxide synthase oxygenase
528191	*Paenibacillus xylanexedens*	BS614_RS00925	3221397 4	nitric oxide synthase oxygenase
421767	*Peribacillus butanolivorans*	AKG34_RS13105	9741035 2	nitric oxide synthase oxygenase
450367	*Peribacillus frigoritolerans*	L8956_RS14020	7236903 2	nitric oxide synthase oxygenase
1349754	*Peribacillus simplex NBRC 15720 = DSM 1321*	BS1321_RS24700	5647591 5	nitric oxide synthase oxygenase
412384	*Priestia aryabhattai*	CR091_RS01875	4801101 7	nitric oxide synthase oxygenase
135735	*Priestia endophytica*	A4R27_RS16730	7276119 4	nitric oxide synthase oxygenase
1121089	*Priestia endophytica DSM 13796*	BM012_RS18755	9371243 7	nitric oxide synthase oxygenase
1402861	*Priestia filamentosa*	B1B01_RS19625	9370332 2	nitric oxide synthase oxygenase
86664	*Priestia flexa*	LZP85_RS00400	9368075 8	nitric oxide synthase oxygenase
1404	*Priestia megaterium*	CE057_RS23090	6414884 8	nitric oxide synthase oxygenase
1348623	*Priestia megaterium NBRC 15308 = ATCC 14581*	EQG57_RS01900	9364072 4	nitric oxide synthase oxygenase
33907	*Pseudonocardia alni*	ATL51_RS16180	9576932 0	nitric oxide synthase oxygenase
172041	*Rhodococcus baikonurensis*	ABWI03_RS08530	9380192 0	nitric oxide synthase oxygenase
1289591	*Rhodococcus erythropolis R138*	H351_RS25075	5748847 3	nitric oxide synthase oxygenase
543736	*Rhodococcus opacus PD630*	K2Z90_RS32625	6989438 2	nitric oxide synthase oxygenase
334542	*Rhodococcus qingshengii*	RQCS_RS06700	6413937 3	nitric oxide synthase oxygenase
1073842	*Rossellomorea aquimaris TF-12*	IQI_RS18970	6774174 4	nitric oxide synthase oxygenase
189381	*Rossellomorea marisflavi*	K6T23_RS03105	8953303 6	nitric oxide synthase oxygenase
218284	*Rossellomorea vietnamensis*	BN987_RS11240	7723690 4	nitric oxide synthase oxygenase
168697	*Salinispora arenicola*	FB564_RS14195	9377206 3	nitric oxide synthase oxygenase
79880	*Shouchella clausii*	SR921_RS06600	8692537 8	nitric oxide synthase oxygenase
985762	*Staphylococcus agnetis*	GJE18_RS07990	5769209 2	nitric oxide synthase oxygenase
985002	*Staphylococcus argenteus*	SAMSHR1132_RS 09360	6684014 2	nitric oxide synthase oxygenase

29378	*Staphylococcus arlettae*	MUA37_RS04535	97287271	nitric oxide synthase oxygenase
93061	*Staphylococcus aureus subsp. aureus NCTC 8325*	SAOUHSC_02134	3921204	
29379	*Staphylococcus auricularis*	I6G39_RS03955	64981781	nitric oxide synthase oxygenase
2742203	*Staphylococcus borealis*	AK212_RS09260	74186796	nitric oxide synthase oxygenase
74703	*Staphylococcus capitis subsp. urealyticus*	V6C80_RS04245	93669080	nitric oxide synthase oxygenase
29380	*Staphylococcus caprae*	JMUB898_RS04870	58050752	nitric oxide synthase oxygenase
1281	*Staphylococcus carnosus*	I6G40_RS03420	93793941	nitric oxide synthase oxygenase
46126	*Staphylococcus chromogenes*	DWB97_RS04255	93655202	nitric oxide synthase oxygenase
74706	*Staphylococcus coagulans*	KM149_RS04360	72413949	nitric oxide synthase oxygenase
29382	*Staphylococcus cohnii*	DYB52_RS04685	58097092	nitric oxide synthase oxygenase
70255	*Staphylococcus condimenti*	EL167_RS04820	93726206	nitric oxide synthase oxygenase
53344	*Staphylococcus delphini*	MUA44_RS04355	77324509	nitric oxide synthase oxygenase
586733	*Staphylococcus devriesei*	DYD94_RS04380	48887559	nitric oxide synthase oxygenase
246432	*Staphylococcus equorum*	I6I25_RS03265	69845455	nitric oxide synthase oxygenase
46127	*Staphylococcus felis*	C7J90_RS11510	48058853	nitric oxide synthase oxygenase
1293	*Staphylococcus gallinarum*	JN168_RS04605	93844716	nitric oxide synthase oxygenase
1283	*Staphylococcus haemolyticus*	EQ029_RS04635	93780423	nitric oxide synthase oxygenase
1290	*Staphylococcus hominis*	EGX58_RS08555	58106903	nitric oxide synthase oxygenase
1284	*Staphylococcus hyicus*	SHYC_RS04160	41072680	nitric oxide synthase oxygenase
29384	*Staphylococcus kloosii*	C7J89_RS05925	69904871	nitric oxide synthase oxygenase
28035	*Staphylococcus lugdunensis*	AL499_RS11665	58091719	nitric oxide synthase oxygenase
214473	*Staphylococcus nepalensis*	BJG89_RS05230	66776406	nitric oxide synthase oxygenase
45972	*Staphylococcus pasteuri*	I6I26_RS04495	72469964	nitric oxide synthase oxygenase
170573	*Staphylococcus pettenkoferi*	CEP67_RS04000	42042983	nitric oxide synthase oxygenase
283734	*Staphylococcus pseudintermedius*	I6G87_RS02695	93823334	nitric oxide synthase oxygenase
2282419	*Staphylococcus pseudoxylosus*	JT704_RS06740	82528862	nitric oxide synthase oxygenase

2282419	*Staphylococcus pseudoxylosus*	LG295_RS05085	97227605	nitric oxide synthase oxygenase
29385	*Staphylococcus saprophyticus*	DV527_RS04545	66867035	nitric oxide synthase oxygenase
342451	*Staphylococcus saprophyticus subsp. saprophyticus ATCC 15305*	SSP_RS04350	3617073	nitric oxide synthase oxygenase
1295	*Staphylococcus schleiferi*	MUA40_RS04150	93789606	nitric oxide synthase oxygenase
2912228	*Staphylococcus shinii*	J5E45_RS13205	79052614	nitric oxide synthase oxygenase
1286	*Staphylococcus simulans*	MUA87_RS04710	77331197	nitric oxide synthase oxygenase
61015	*Staphylococcus succinus*	PYW31_RS04395	93720032	nitric oxide synthase oxygenase
94138	*Staphylococcus ureilyticus*	MUA21_RS08330	78332759	nitric oxide synthase oxygenase
1292	*Staphylococcus warneri*	D3P10_RS04555	58059673	nitric oxide synthase oxygenase
1288	*Staphylococcus xylosus*	SXYLSMQ121_RS04585	45496551	nitric oxide synthase oxygenase
42234	*Streptomyces acidiscabies*	J8M50_RS30930	69810397	nitric oxide synthase oxygenase
1893	*Streptomyces atratus*	C5746_RS05260	95517924	nitric oxide synthase oxygenase
227882	*Streptomyces avermitilis MA-4680 = NBRC 14893*	SAVERM_RS08125	41538628	nitric oxide synthase oxygenase
2746961	*Streptomyces caniscabiei*	IHE65_RS14920	79930761	nitric oxide synthase oxygenase
67260	*Streptomyces cinereoruber*	CP977_RS31910	95458368	nitric oxide synthase oxygenase
285574	*Streptomyces erythrochromogenes*	OHA91_RS32925	95500954	nitric oxide synthase oxygenase
67294	*Streptomyces filamentosus*	CP979_RS29005	95662483	nitric oxide synthase oxygenase
66892	*Streptomyces gardneri*	H4W23_RS13080	95689506	nitric oxide synthase oxygenase
1942	*Streptomyces goshikiensis*	PET44_RS03740	91407980	nitric oxide synthase oxygenase
1942	*Streptomyces goshikiensis*	PET44_RS05155	91408263	nitric oxide synthase oxygenase
1873719	*Streptomyces hydrogenans*	AB0O27_RS11355	94006858	nitric oxide synthase oxygenase
58340	*Streptomyces lavendulae subsp. lavendulae*	SLAV_RS32120	49387430	nitric oxide synthase oxygenase
1535768	*Streptomyces lunaelactis*	SLUN_RS05280	55654673	nitric oxide synthase oxygenase
68239	*Streptomyces mirabilis*	OG981_RS11240	93996070	nitric oxide synthase oxygenase
66374	*Streptomyces nojiriensis*	JYK04_RS07570	95588462	nitric oxide synthase oxygenase
67358	*Streptomyces roseolus*	IE245_RS28705	95531246	nitric oxide synthase oxygenase

680198	*Streptomyces scabiei 87.22*	SCAB_RS15100	24308273	nitric oxide synthase oxygenase
146820	*Streptomyces stelliscabiei*	H4687_RS30490	86830664	nitric oxide synthase oxygenase
67367	*Streptomyces tanashiensis*	LDH80_RS35840	95604923	nitric oxide synthase oxygenase
85558	*Streptomyces turgidiscabies*	PV735_RS46140	97407478	nitric oxide synthase oxygenase
1048205	*Streptomyces uncialis*	OG711_RS34025	96796522	nitric oxide synthase oxygenase
953739	*Streptomyces venezuelae ATCC 10712*	DEJ43_RS32895	51867098	nitric oxide synthase oxygenase
1960	*Streptomyces vinaceus*	CP980_RS29130	95614623	nitric oxide synthase oxygenase
1961	*Streptomyces virginiae*	JOF58_RS06835	86952959	nitric oxide synthase oxygenase
1196353	*Streptomyces wuyuanensis*	BLT82_RS14065	40830125	nitric oxide synthase oxygenase
67384	*Streptomyces xanthochromogenes*	IE233_RS38585	96295693	nitric oxide synthase oxygenase
67385	*Streptomyces xanthophaeus*	KPP03845_RS06250	96801939	nitric oxide synthase oxygenase
1648182	*Streptomyces yangpuensis*	NRK68_RS04255	95572654	nitric oxide synthase oxygenase
68286	*Streptomyces zaomyceticus*	OG567_RS34465	97201843	nitric oxide synthase oxygenase
324952	*Streptosporangium canum*	BM085_RS17040	96299151	nitric oxide synthase oxygenase
79883	*Sutcliffiella horikoshii*	B4U37_RS04205	96737630	nitric oxide synthase oxygenase
361277	*Terribacillus saccharophilus*	P4693_RS07075	93918430	nitric oxide synthase oxygenase
1482	*Virgibacillus halodenitrificans*	BME96_RS02665	71513280	nitric oxide synthase oxygenase
1473	*Virgibacillus pantothenticus*	KBP50_RS13320	66871405	nitric oxide synthase oxygenase

Data available in: https://www.ncbi.nlm.nih.gov/sites/myncbi/ignacio andres.jofre fernandez.1/collections/64872640/public

Supplementary Figure S1

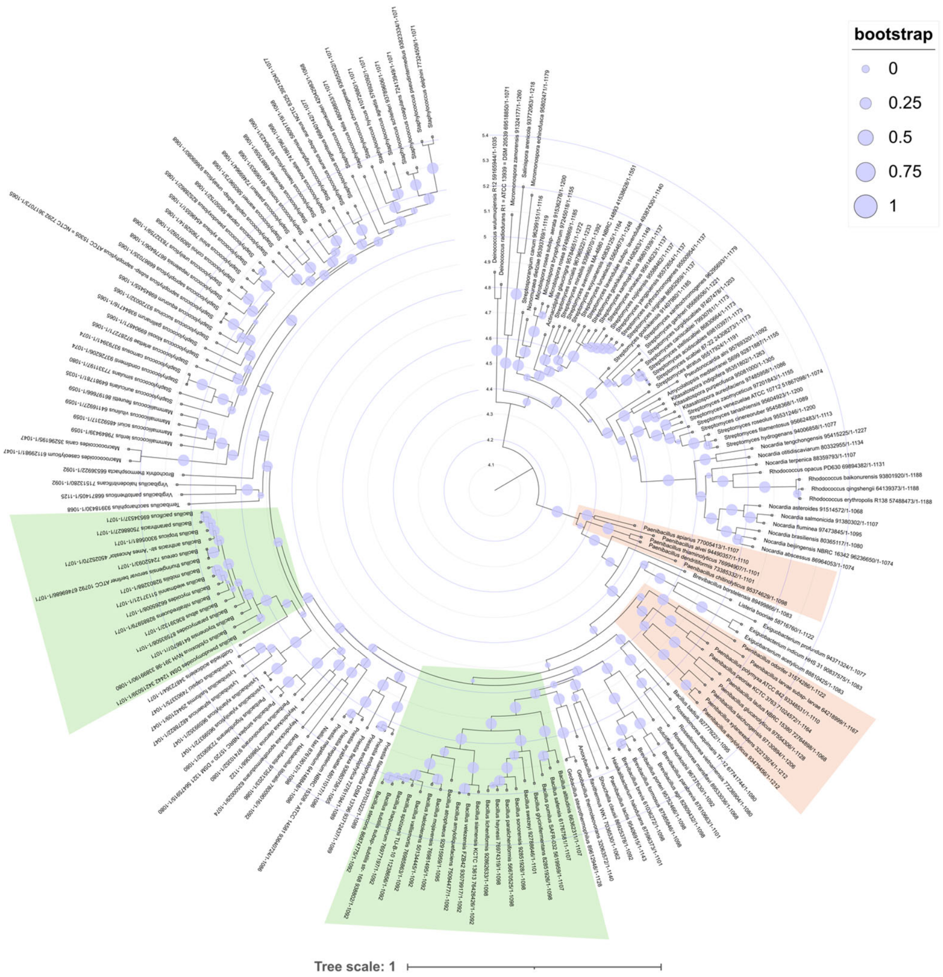

Figure S1. Phylogenetic analysis of bacterial nitric oxide synthase (*bnos*) genes. The phylogenetic relationships of *bnos* genes identified in bacterial genera. Sequences were retrieved from the GenBank database and aligned using MAFFT. Bootstrap values (ranging from 0 to 1) are indicated by circle sizes, representing the confidence of each branch. Colored clades highlight *Paenibacillus* (green) and *Bacillus* (orange) sequences, which were the most genera-convergent based on homology. This analysis guided the identification of conserved regions for primer design, targeting six high-homology regions across these genera.

Supplementary Table S2

Table S2. Organisms used as reference for *bnos* primer design. Data available in: https://www.ncbi.nlm.nih.gov/sites/myncbi/ignacio andres.jofre fernandez.1/collections/64872640/public/

Accession number of genomic regions	Species	Gene ID
NC_000964.3	*Bacillus subtilis* subsp. *subtilis*	*938802*
NZ_CP007640.1	*Bacillus atrophaeus* subsp. *globigii*	*92915959*
NZ_CP034943.1	*Bacillus spizizenii*	*11238656*
NZ_CP072120.1	*Bacillus amyloliquefaciens*	*75094477*
NZ_CP033052.1	*Bacillus vallismortis*	*76985863*
NZ_CP051464.1	*Bacillus mojavensis*	*76981495*
NZ_CP009679.1	*Bacillus velezensis*	*93079917*
NZ_CP096592.1	*Bacillus inaquosorum*	*76977197*
NZ_CP029364.1	*Bacillus halotolerans*	*50134445*
NZ_AOFM01000005.1	*Bacillus sonorensis*	*92851928*
NZ_CP049019.1	*Bacillus tropicus*	*93005681*
NZ_CM000753.1	*Bacillus thuringiensis*	*67469686*
NZ_CM000745.1	*Bacillus pseudomycoides*	*34215039*
NZ_AGFX01000018.1	*Paenibacillus peoriae*	*71024572*
NZ_AP025344.1	*Paenibacillus dendritiformis*	*73385332*
NZ_CP019659.1	*Paenibacillus larvae subsp. larvae*	*64218999*
NZ_CP041405.1	*Paenibacillus thiaminolyticus*	*76994907*
NZ_CP009428.1	*Paenibacillus odorifer*	*31574286*
NZ_CP024795.1	*Paenibacillus polymyxa*	*93348531*
NZ_JAGGKI010000018.1	*Paenibacillus lactis*	*95406915*
NZ_CP026520.1	*Paenibacillus chitinolyticus*	*95374629*
NZ_JAOQAR020000006.1	*Paenibacillus alvei*	*94490357*
NZ_CP145892.1	*Paenibacillus amylolyticus*	*93479456*
NZ_JAFFHZ010000002.1	*Paenibacillus apiarius*	*77005413*

Supplementary Figure S2

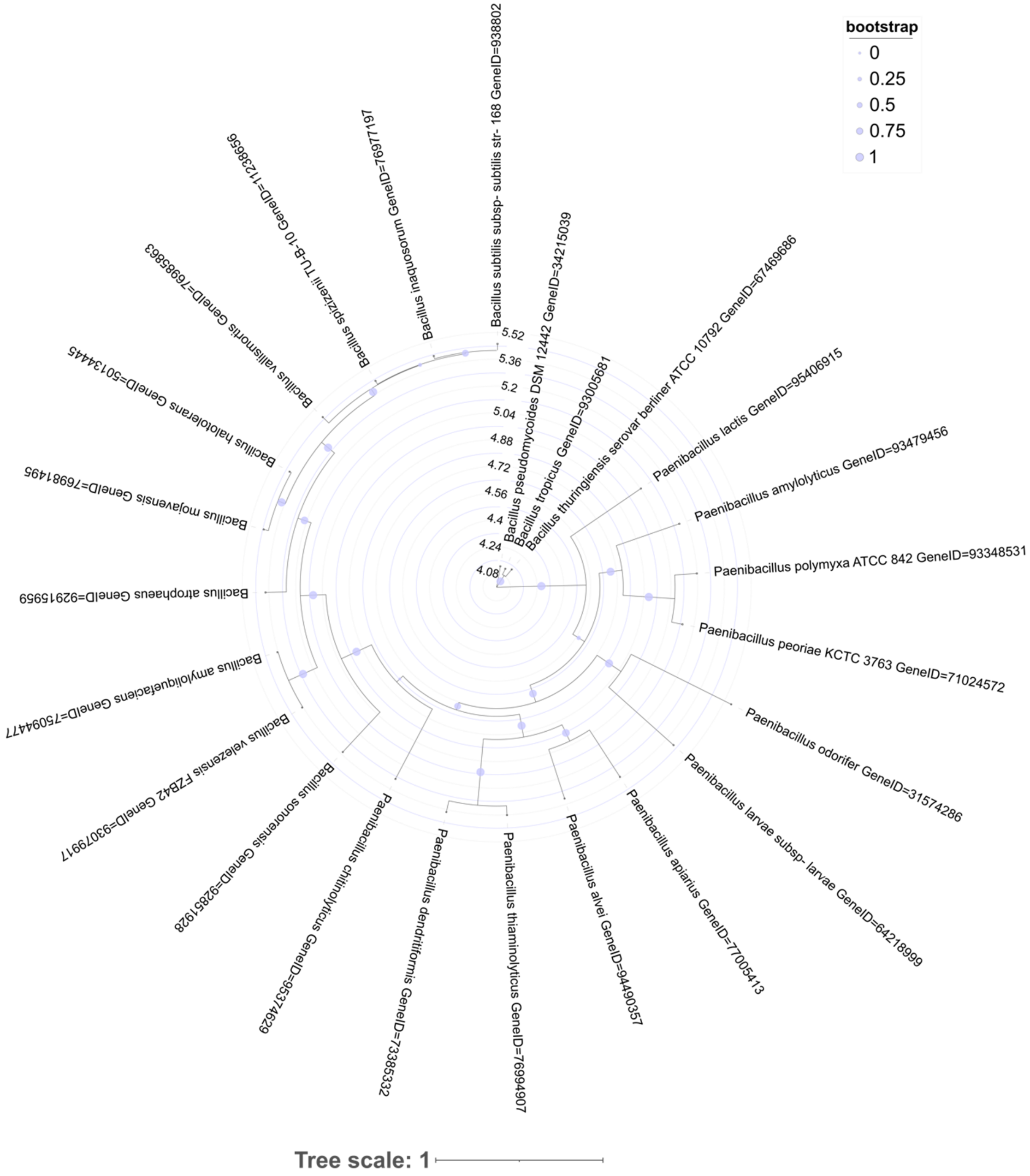

Figure S2. Phylogenetic similarity of bacterial nitric oxide synthase (*bnos*) genes in selected genus. The phylogenetic relationships of aligned *bnos* genes identified in *Bacillus* and *Paenibacillus* genus. Bootstrap values (ranging from 0 to 1) are indicated by circle sizes, representing the confidence of each branch.

Supplementary Table S3

Table S3. Pairwise dDDH values between strain AC7 and TYGS genomes. The dDDH values are provided along with their confidence intervals for the three different GBDP formulas.

Query strain	Subject strain	% dDDH (d0)	% dDDH (d4)	% dDDH (d6)	G+C difference (%)
Strain AC7	*P. pseudetheri*	61,3	53,7	61	0,29
	P. etheri	64,9	48	62,5	0,2
	P. odorifer	52,7	30,1	46,1	0,46
	P. tianjinensis	17,1	20,3	16,9	5,77
	P. wynnii	16,7	20,3	16,6	1,12
	P. agri	17,1	20,3	16,9	4,33
	P. borealis	16,7	20,2	16,6	7,64
	P. riograndensis	16,1	20,2	16,1	7,22
	P. albidus	17	20,1	16,8	6,13
	P. jilunlii	16,3	20	16,2	7,13
	P. phytohabitans	16,3	20	16,2	7,32
	P. silagei	15,7	19,7	15,7	8,75
	P. rhizoplanae	15,6	19,7	15,6	8,39
	P. camerounensis	15,9	19,3	15,9	7,62

•d0: length of all high-scoring segment pairs (HSPs) divided by total genome length

•d4: sum of all identities found in HSPs divided by overall HSP length

•d6: sum of all identities found in HSPs divided by total genome length

Supplementary Figure S3

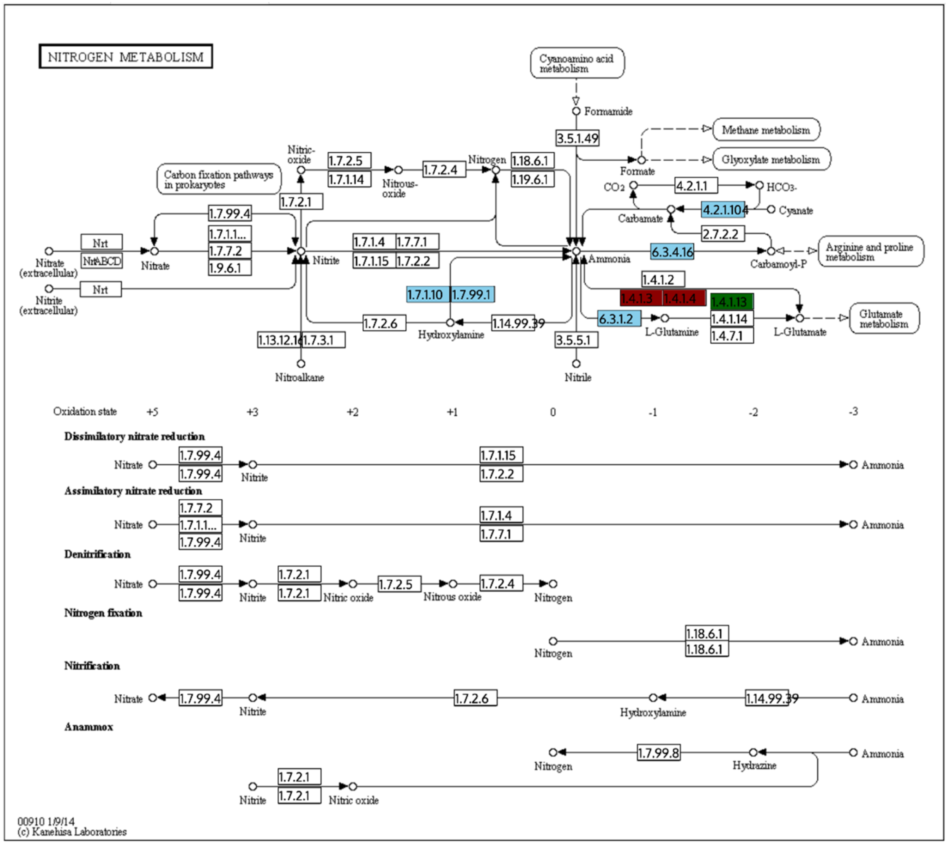

Figure S3. Nitrogen Metabolism pathway in strain AC7. Reactions presents in blue, with positive fluxes displayed in shades of red and negative fluxes in shades of green. In blue: 1.7.1.10 hydroxylamine reductase ($NADH_2$), 1.7.99.1 hydroxylamine reductase, 6.3.4.16 carbamoyl-phosphate synthase (ammonia), 4.2.1.104 cyanate lyase, and 6.3.1.2 glutamine synthetase. In red: 1.4.1.3 and 1.4.1.4; glutamate dehydrogenase ($NAD(P)^+$)). In green: 1.4.1.13 glutamate synthase (NADPH) large chain

Supplementary File S1.

File S1. Nucleotide alignment of *bnos* gene sequences and BLASTn results. Multiple sequence alignment (MSA) of *bnos* nucleotide sequences from *Paenibacillus nitricinens* strain AC7 and related taxa, used for phylogenetic analysis. The alignment was generated using MAFFT and formatted as a FASTA file.

Supplementary File S2.

File S2. Amino acid alignment of bNOS protein sequences and BLASTp results. Multiple sequence alignment (MSA) of deduced bNOS amino acid sequences from *Paenibacillus nitricinens* strain AC7 and reference organisms. The alignment was generated using MAFFT and formatted in FASTA for phylogenetic reconstruction. BLASTp results against the NCBI nr database are also included, confirming the functional classification of the protein as a nitric oxide synthase oxygenase homolog.
